# The influence of skin colour on the experience of ownership in the rubber hand illusion

**DOI:** 10.1038/s41598-017-16137-3

**Published:** 2017-11-16

**Authors:** Marilia Lira, Julia H. Egito, Patricia A. Dall’Agnol, David M. Amodio, Óscar F. Gonçalves, Paulo S. Boggio

**Affiliations:** 1 Social and Cognitive Neuroscience Laboratory and Developmental Disorders Program, Center for Health and Biological Sciences, Mackenzie Presbyterian University, 01241-001 Sao Paulo, Brazil; 2 Neuropsychophysiology Lab, CIPsi, School of Psychology, University of Minho, Braga, Portugal; 3 Spaulding Center of Neuromodulation, Department of Physical Medicine & Rehabilitation, Spaulding Rehabilitation Hospital and Massachusetts General Hospital, Harvard Medical School, Boston, MA USA; 4 Department of Psychology, University of Amsterdam, Amsterdam, NL & Department of Psychology, New York University, NY, USA

## Abstract

Racial prejudice is associated with a fundamental distinction between “us” and “them”—a distinction linked to the perceived overlap between representations of the self and others. Implicit prejudice has been shown to reduce the intensity of White individuals’ hand ownership sensation as induced by the Rubber Hand Illusion (RHI) with dark rubber hands. However, evidence for this link to implicit prejudice comes from self-report questionnaire data regarding the RHI. As an alternative, we assessed the onset time of the RHI. We hypothesized that onset time of the RHI would be higher for the black compared to the white RH, acting as the mediator between implicit prejudice and magnitude of the RH illusion and proprioceptive drift. As expected, participants took longer to incorporate the black RH and presented lower RH illusion magnitude and a smaller proprioceptive drift for the black RH. Mediation analysis revealed a significant indirect effect of implicit racial bias on proprioceptive drift and magnitude of illusion through onset time to illusion only for the black RH. These findings further illuminate the connection between implicit prejudice and embodied perception, suggesting new perspectives on how implicit biases operate.

## Introduction

Racial prejudice is associated with a fundamental distinction between “us” and “them”—a distinction that has been linked to the degree of perceived overlap between cognitive representations of the self and others^1,2^. Recent findings have shown that this distinction extends to perceptions of physical overlap, such that prejudices may reside in one’s bodily representations, in addition to their cognitive representations^3,4^. These findings suggest the possibility that prejudices may also be represented in the extent to which our racial identity is ingrained in our body awareness, whereby racial prejudice relates to stricter distinctions between physical representations of the self and racial outgroup members.

Body awareness results from subjectively experiencing a physical body as the product of a multisensory integration from different modal systems^5–7^. Previous studies have investigated the modulation of subjective body experience using the Rubber Hand visual-tactile illusion^6^ (RHI). RHI is a multisensory experience that simultaneously integrates visual and tactile stimuli to induce a sense of ownership of a realistic looking rubber hand (RH). In this illusion, the RH is located inside the peripersonal space in a position congruent with the participant’s hand. During the RHI, participants are asked to look at a paintbrush stimulating the RH while another paintbrush synchronously stimulates the participant’s hand^6,8^. In the course of RHI, the majority of the participants report experiencing the RH as their own hand^9–11^.

Research has begun to examine how different characteristics of the RH (e.g. size, gender, color) modulate the degree of the RHI effect. Skin color, a crucial prototypic feature for distinguishing social group identity, has been used in studies addressing ingroup/outgroup categorization^12–15^. To the best of our knowledge, only three studies have examined the effects of skin color on the RHI^12,16,17^. Farmer *et al*.^12^ observed that White participants felt the RHI to some degree regardless of the hand’s color (white versus black). Moreover, baseline Implicit Association Task (IAT) did not predicted illusion effects. However, the self-reported sensation of hand ownership, as assessed by the illusion questionnaire, was more intense with the white RH compared with the black RH. Furthermore, the authors found a negative correlation between illusion intensity and participants’ implicit racial bias assessed after the RHI, suggesting that a stronger experience of the RH illusion across color conditions was associated with lower implicit prejudice. In a similar vein, Maister *et al*.^17^ reported that the ownership experience of a dark RH in white participants altered the implicit racial bias; i.e. greater subjective experience of hand ownership resulted in lower implicit bias. This pattern is consistent with the findings of Peck *et al*.^15^, in which the perceptual appropriation of a dark body avatar was associated with a decrease in White participant’s implicit racial bias scores.

The three studies described above^12,15,17^ used self-report questionnaires to assess the magnitude of the illusion and Farmer *et al*.^12^ also assessed the proprioceptive drift of the participant’s hand towards the RH. However, no studies have used the RHI *time to onset*—that is, participants’ report on the exact time they start experiencing the RHI—as a strategy for uncovering potential racial biases. The time to onset of the RHI may be understood as the period required for achieving multisensory integration. The lower the RHI time to onset, the faster the multisensory integration. By contrast, longer times to RHI onset may be related to difficulties in creating a unique percept from different sensorial inputs. Therefore, the lack of effects observed between baseline racial bias and the illusion could have been due to not linking timing with magnitude of the illusion. As timing is an important component in multisensory integration, measuring the onset time to illusion might reveal previously-obscured phenomena that link racial bias and body ownership.

The aim of the present study was to investigate the effect of the skin color (black versus white) on the onset of the RHI in White participants by testing whether the onset time was related to the intensity of the illusion (Rubber Hand Illusion Questionnaire and Proprioceptive Drift Test), and whether these effects are predicted by implicit racial bias (Implicit Association Test). We hypothesized an increase in the RHI onset time in the black RH condition. Moreover, we expected that the RHI time to onset would act as a mediator between implicit racial bias and the RHI effect, as indexed by either the magnitude of the illusion or proprioceptive drift. This mediation hypothesis is based on the assumption that participants with higher IAT scores will need to engage a greater degree of multisensory processing of the other-race RHI (resulting in an increase of the onset time to illusion) and this effect will result in a less robust illusion (as indexed by the magnitude of the illusion and the proprioceptive drift).

## Method

### Participants and design

Ninety-five white university students participated in exchange for extra course credit. Participants were recruited for the study by requesting white volunteers by the local university. Participants’ self-identification as white people was further confirmed by an interview requesting further information about their ancestry. Participants reported no African, Asian or Native American ancestry. All were right-handed as assessed by the Edinburgh Handedness Inventory^18^ and none had previously experienced the RHI as well as any history of neurological disorders, disorders related to body image, or burns or nerve damage to the upper limbs. The study was approved by the Institutional Review Board of the Mackenzie Presbyterian University and by the National Ethics Committee (SISNEP, Brazil), all participants provided written informed consent, and all experiments were performed in accordance with relevant guidelines and regulations.

### Design

Participants were assigned to either a synchronous (N = 48, 38 female) or asynchronous (N = 47, 36 female) stimulation condition. In each condition, RHI was assessed for both a black and white hand. Thus, the design was a 2 (synchronous vs asynchronous) × 2 (black vs. white hand) mixed factorial, with repeated measures on hand color. It is notable that two previous studies examining black and white RHI used somewhat different experimental designs. Farmer *et al*.^12^ used a within-subjects design, whereas Maister *et al*.^17^ used a between-subjects design. Their designs are justified by their main goals: the experience of RHI with different RH colors and the effect of different RH colors on the implicit bias, respectively. However, in the present research, a mixed-model design, with RH color as a within factor and synchronous/asynchronous stimulation, was deemed most more appropriate given our specific questions and hypotheses. Particularly, it was important to have a within-subjects comparison of White vs. Black RHI in order to make a more direct comparison with participants IAT scores, which are based on White-Black comparisons.

### RHI Apparatus

The RHI apparatus was specifically designed for the objectives of this study (for technical details, contact the corresponding author). The apparatus (103 cm wide by 51 cm tall) includes two motorized pulley systems (one fixed and one movable) corresponding to the arms moving the paintbrushes. The movable pulley system allows adjustment for different hand sizes. On the outside, the apparatus had a control panel and a microprocessor regulating the movement of the paintbrushes (i.e., angle, speed and distance). The equipment allows the possibility of reversing the direction of paintbrushes movement (e.g., to generate an asynchronous stimulation) as well as the possibility of manual or automatic lateral shifting (e.g., stimulation of the whole hand). All functions were handled from the control panel by binary command keys creating multiple combinations of speed, angle, and distance. The trolley slide distance was controlled by the position of two tabs composed of mobile optical switches and fixed by magnets. In the synchronous stimulation condition, the paintbrushes were moved in the same direction to provide a synchronic tactile stimulation. In the asynchronous stimulation condition, the paintbrushes were moved in opposite directions. The angular velocity was 158 ms/mm with an angle of 11 degrees. The paintbrush stimulation was limited to the first two phalanges of the index finger of the left hand. The efficacy of the current RHI apparatus in generating the RHI was confirmed in a previous study^19^.

### RH Prostheses

**Figure 1:**
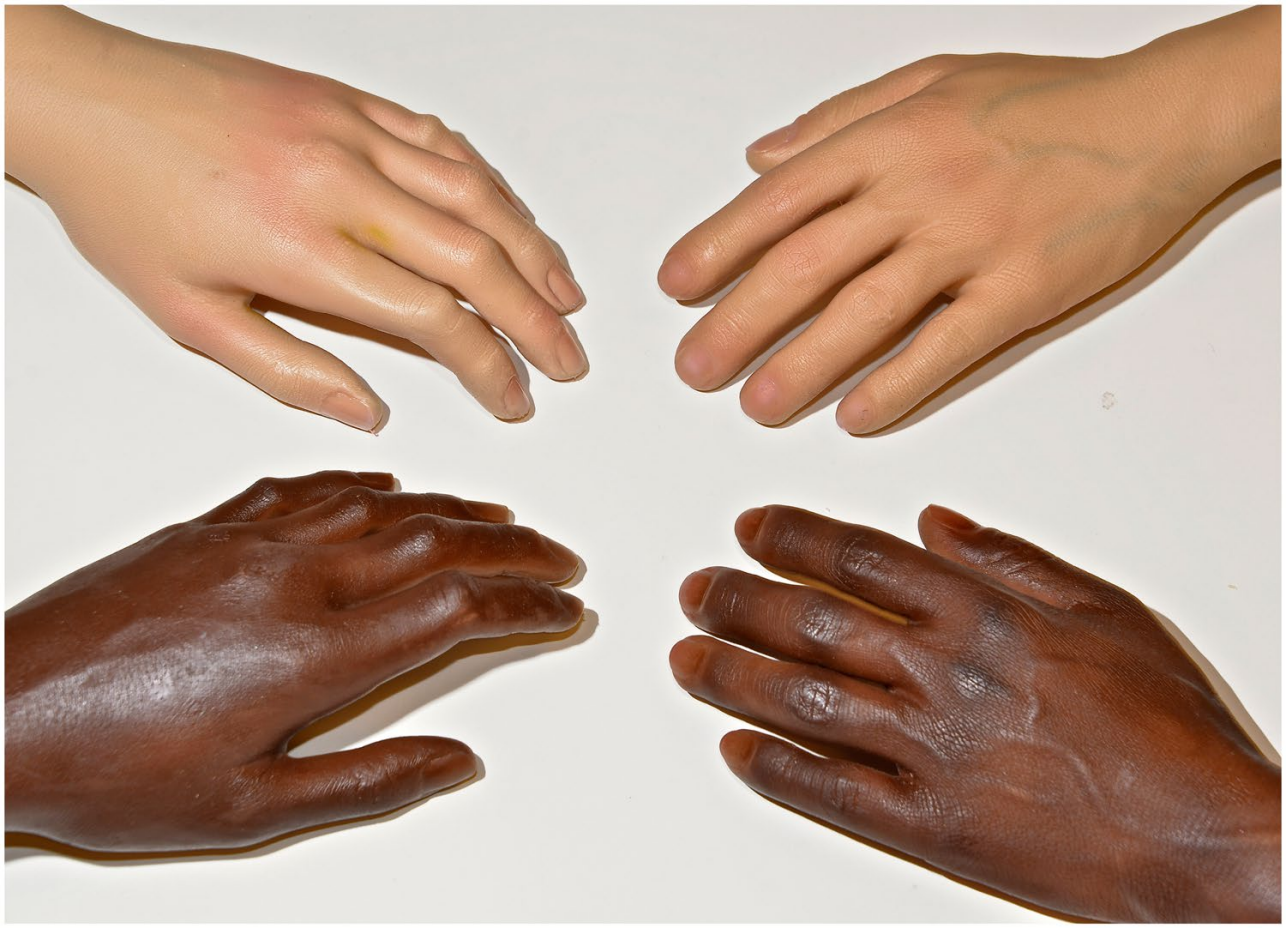
Black and white, male and female rubber hands.

RH prostheses were specifically made for this study. We used four prostheses of left hand and forearm including combination of color (black- and white-skinned) and gender (female and male) (see Fig. 1). All participants completed the RHI protocol with the same-gender prostheses of both colors.

### Proprioceptive Drift Test

In order to measure proprioceptive drift, participants were instructed to close their eyes and try to localize (without touch, just pointing) with their right index finger where they thought their left index finger was localized. This measure was collected before and after the RHI. The proprioceptive drift index was calculated by the difference between post and pre RHI measures^20,21^. Positive higher proprioceptive drift means that the drift was toward the rubber hand (from the real hand). A proprioceptive drift index equal zero means that no drift happened after the RHI. Finally, a negative proprioceptive drift index means that a drift away from the rubber hand.

### Onset Time to the RHI

Initially, the participants were informed that during the experiment they could have the sensation that the rubber hand was their own hand and they should report the exact moment when they start feeling the RHI (i.e., “I am feeling it”). Time count started at the beginning of paintbrushes stimulation and stopped as soon as the participant reported the sensation (i.e., “I am feeling it”). Onset time to illusion was registered by the experimenter with the use of a stopwatch.

### Rubber Hand Illusion Questionnaire

The magnitude of the RHI was assessed using a self-report questionnaire (adapted from Botvinick & Cohen^6^ and Longo *et al*.^20^). This measure consisted of seven statements: (1) “It seemed like I was looking directly at my own hand, rather than at a rubber hand,” (2) “The rubber hand began to resemble my own (real) hand,” (3) “It seemed like the rubber hand belonged to me,” (4) “I felt as if the rubber hand were my hand,” (5) “It seemed like the rubber hand was part of my own body,” (6) “It seemed like the rubber hand was in the location where my hand was,” and (7) “It seemed like the touch I felt was caused by the paintbrush touching the rubber hand.” Reponses to each statement were provided in an analog Likert scale ranging from 1 to 5 (1- totally disagree; 5 - totally agree) and an average score was computed for each participant.

### Implicit Association Test (Race IAT)

In order to better understand the role of implicit prejudice on the RHI, participants completed the race Implicit Association Test (IAT) developed by Greenwald, McGhee & Schwartz^22^. The race IAT consists of the presentation of five blocks of trials (20 trails each) in which participants classify Black and White male faces by race and positive (pleasant, happy, joy and friendly) or negative (angry, disgust, awful and bad) valence. This task includes two critical blocks of trials, in which these two classification schemes are combined. In the “compatible” block, participants used one key to classify positive words and White faces, and another key to classify negative words and Black faces. In the “incompatible” block, participants used one key to classify positive words and Black faces, and another key to classify negative words and White faces. Thus, to the extent an individual possess anti-Black/pro-White associations, classifications would be easier, and hence quicker, on compatible than incompatible blacks. Participants were instructed to perform the task as quickly as possible. A Portuguese version of the IAT was used in this study. Responses were scored using the *D* statistic, which represents the difference in response latency between incompatible and compatible blocks, divided by their pooled standard deviations^23,24^.

### Interpersonal Reactivity Index (IRI)

To examine the role of empathy in the RHI, participants completed the Interpersonal Reactivity Index^25^ (IRI; as adapted by Sampaio, Guimarães, Camino, Formiga e Menezes^26^). The IRI includes 28 items and four subscales (perspective taking, fantasy, empathic concern, and personal distress). Responses were made on 5 point Likert scale (0 = does not describe me well to 4 = describe me well).

### Procedure

Participants who met inclusion/exclusion criteria were invited to the laboratory two times with a 24-hour interval. After signing the consent term and providing demographic and health information, participants’ hand laterality was assessed by the Handedness Inventory of Edinburgh followed by the IAT and IRI.

After receiving instructions about the experimental procedure, participants were invited to sit comfortably in a chair with knees and ankles at 90° angles, feet on the floor, and arms resting on a table (measures 90 × 50 × 35) with elbows bent at 90 degrees. The participants’ left hand rested on the table and a wooden partition occluded it from view. The RH replaced the natural positioning of the participant’s hand at the visible side of the wooden partition, with a distance of 17 cm between the forefingers of the RH and the participants’ left hand^27^. Participants were instructed not to move during the entire RHI protocol.

**Figure 2:**
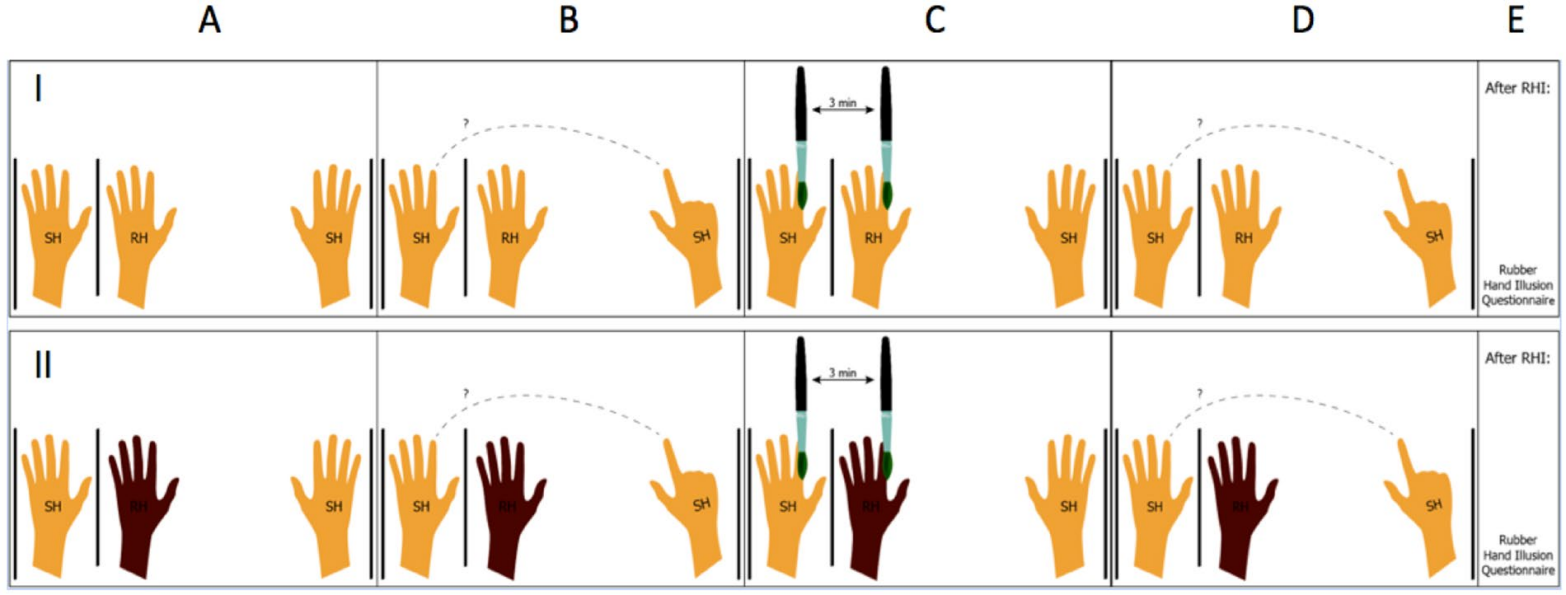
Two condition of RHI: (I) white RH and (II) black RH. (**A**) The RH was positioned parallel to the participant’s hand (SH). (**B**) A first measure of proprioceptive drift was made. (**C**) RHI was induced by the visuo-tactile stimulation with white RH (I) and black RH (II) and the participant was asked to report when he/she started feeling the illusion. (**D**) After 3 minutes of visuo-tactile stimulation, a second measure of the proprioceptive drift was collected. (**E**) Finally, the participant was requested to respond to the RHI questionnaire.

After positioning, participants were asked to close their eyes and perform the proprioceptive drift test. Then, the experimenter placed the wooden partition between the participants’ hand and the RH, blocking the view of their own hand. Participants were instructed to maintain view of the RH as the visuo-tactile stimulation was initiated, and to report the exact moment when they started perceiving the illusion (i.e, Onset Time to the RHI). The visuo-tactile stimulation last for 3 minutes. After the RHI protocol, a second measure of the proprioceptive drift was collected (with both eyes closed). Finally, participants completed the RHI Questionnaire (see Fig. 2). At each run (separated by a 24-hour interval), the experimental procedures took place with different RH color in a randomized and counterbalanced order.

### Data Availability

The datasets generated during and/or analyzed during the current study are available from the corresponding author on reasonable request.

## Results

### Demographic data

As shown in Table 1, participants in both conditions exhibited a strong degree of implicit bias in favor of white individuals. IRI scores were in the mean range when compared to normative scores^26^. Additionally, participants from the synchronous and asynchronous conditions did not differ significantly in terms of age, IAT score, perspective taking, empathic concern, or fantasy. However, the synchronous group presented increased reports of personal distress when compared with the asynchronous group.

**Table 1:** Demographic data.

	Synchronous		Asynchronous		*t*	*P*
	Mean	±S.E.	Mean	±S.E.		
Age (years)	23.60	±0.93	22.09	±0.66	1.33	0.19
IAT (d score)	0.74	±0.05	0.62	±0.06	1.46	0.15
Perspective Taking	19.58	±0.38	18.55	±0.48	1.70	0.09
Empathic Concern	17.02	±0.29	17.96	±0.50	−1.63	0.11
Fantasy	18.57	±0.53	17.62	±0.61	1.18	0.24
Personal Distress	18.72	±0.52	14.98	±0.69	4.33	<0.0001

To address if the significant difference on personal distress between conditions could explain our findings on RH measures, analyses of covariance with personal distress as a continuous predictor were performed for the onset time to illusion, proprioceptive drift and questionnaire. These analyses did not reveal any significant effect (p > 0.05).

### Onset Time of the RHI

**Figure 3:**
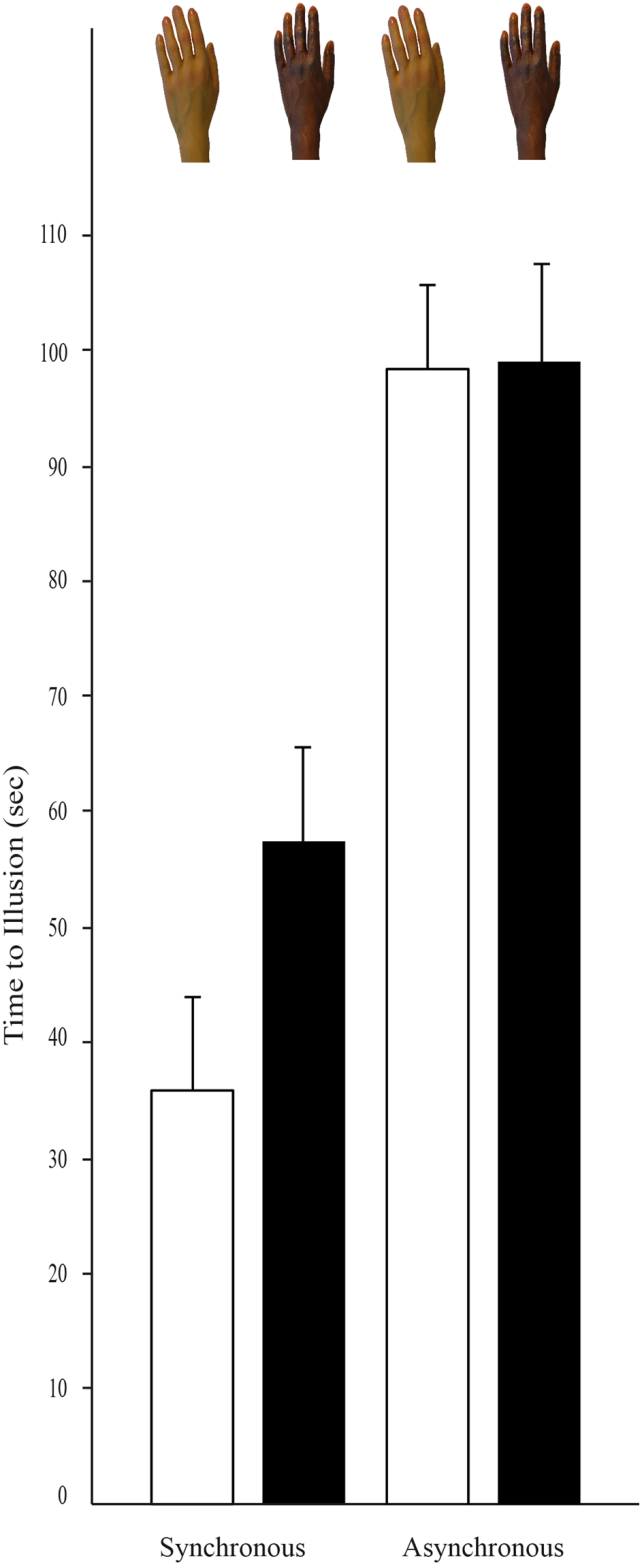
Mean time to illusion by the RH color and by stimulation condition (synchronous and asynchronous). Error bars represent SEs.

To compare the onset time of the RHI between the black and white RH in both synchronous and asynchronous conditions, we performed a mixed model ANOVA with order of hand color use (black first vs. white first) and stimulation condition (synchronous vs. asynchronous) as between-subjects factors and hand color (Black vs. White) as the within-subjects factor. This ANOVA revealed significant effects for stimulation condition, *F*(1,91) = 8.55, *p* = 0.004, η2 = 0.09, and rubber hand color, *F*(1,91) = 4.56, *p* = 0.04, η2 = 0.05, which were qualified by the predicted interaction, *F*(1,91) = 4.39, *p* = 0.04, η2 = 0.05 (Fig. 3). Simple effect analyses revealed that the onset time of the illusion for the white rubber hand during the synchronous condition was smaller when compared to black RH, *t*(47) = 3.28, *p* = 0.002, but no significant difference was found between white and black RH during the asynchronous condition, *t*(46) = −0.03, *p* = 0.98. Significant effects did not emerge for order, *F*(1,91) = 1.66, *p* = 0.20, η2 = 0.02, the interaction of order and stimulation condition, *F* (1,91) = 1.19, *p* = 0.28, η2 = 0.01, the interaction order and rubber hand color, *F*(1,91) = 0.06, *p* = 0.81, η2 = 0.0006, or the three-way interaction, *F*(1,91) = 0.57, *p* = 0.45, η2 = 0.006.

#### Proprioceptive Drift

**Figure 4:**
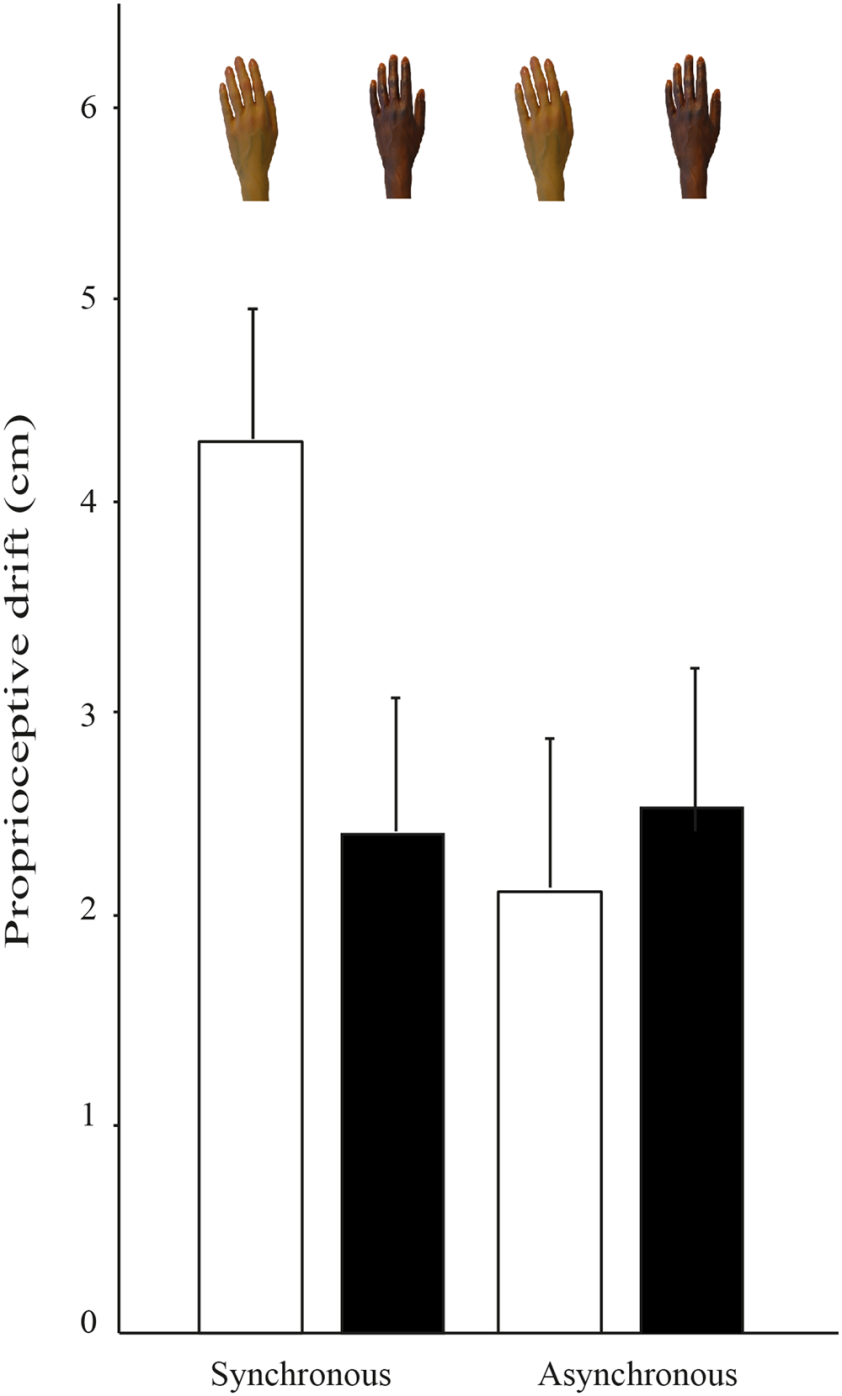
Mean proprioceptive drift by the RH color separated by stimulation condition (synchronous and asynchronous). Error bars represent SEs. Positive proprioceptive drift refers to the degree of drift toward the rubber hand after the illusion relative to baseline. On the y-axis, zero indicates no difference between the pre and post measures of drift.

To compare the proprioceptive drift induced by the black and white RH in both synchronous and asynchronous conditions, we performed a mixed model ANOVA as above. This analysis produced a significant Stimulation Condition X Rubber Hand Color interaction, *F*(1,91) = 4.84, *p* = 0.03, η2 = 0.05. As shown in Fig. 4, simple effect tests revealed that the proprioceptive drift for the white RH during the synchronous condition was greater than the drift for the black RH, *t*(47) = −2.38, *p* = 0.02, but no significant difference was found between white and black RH during the asynchronous condition, *t*(46) = 0.43, *p* = 0.67). Additionally, no significant effects were found for stimulation condition, *F*(1,91) = 1.63, *p* = 0.21, η2 = 0.02, rubber hand color, *F*(1,91) = 2.13, *p* = 0.15, η2 = 0.03, order, *F*(1,91) = 0.11, *p* = 0.74, η2 = 0.001, the interaction of order and stimulation condition, *F*(1,91) = 0.02, *p* = 0.89, η2 = 0.0002, the interaction order and rubber hand color, *F*(1,91) = 0.26, *p* = 0.61, η2 = 0.003, or the three-way interaction, *F*(1,91) = 3.08, *p* = 0.08, η2 = 0.03.

#### Rubber Hand Illusion Questionnaire

**Figure 5:**
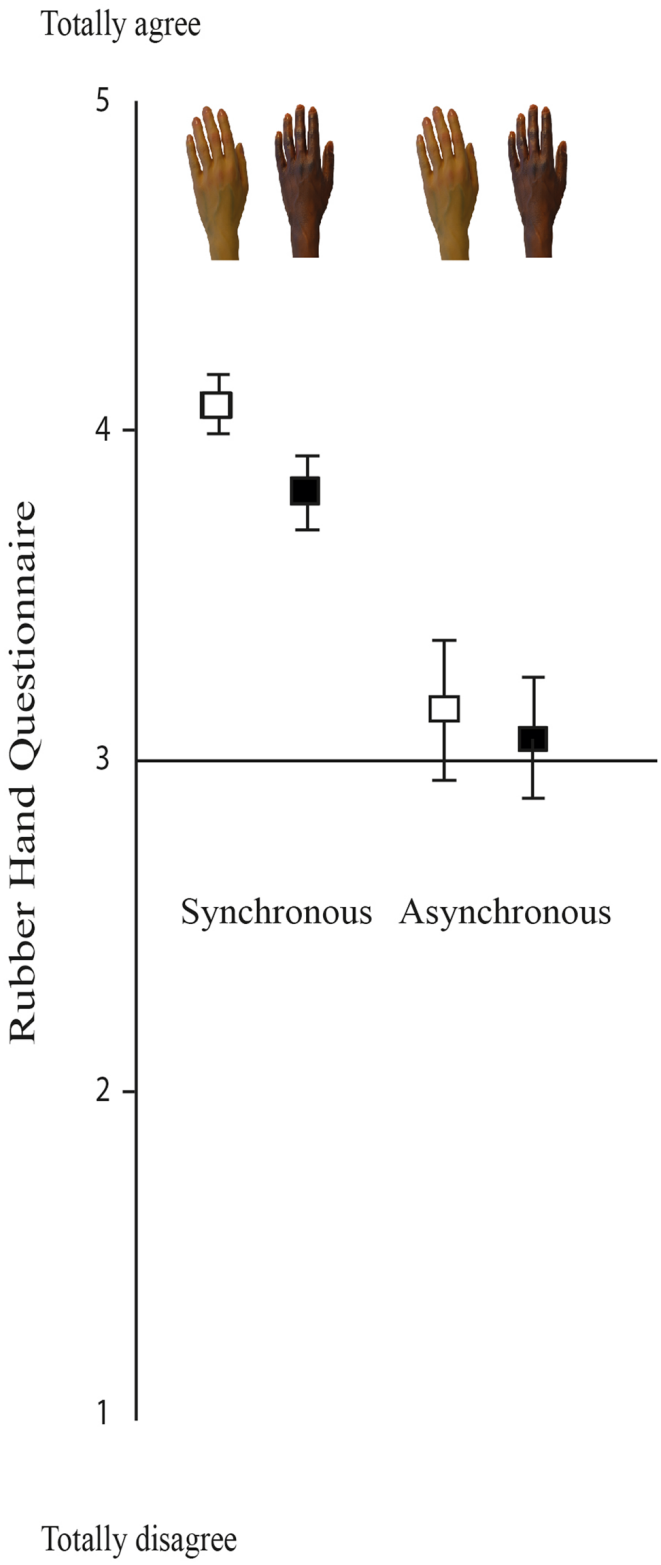
Mean and S.E. of all questions of the Rubber Hand Illusion Questionnaire by the RH color.

Scores on the RHI questionnaire were tested using the mixed model ANOVA as above. ANOVA revealed main effects for condition, F(1,91) = 13.38, *p* < 0.001, η2 = 0.13, indicating a stronger RHI effect in the synchronous condition as compared to the asynchronous condition (Fig. 5). Follow-up analyses confirmed that scores in the synchronous condition were significantly above the scale midpoint (i.e., a score of 3: *do not agree nor disagree*), using one-sample *t*-tests, indicating a strong reported RHI for both the white (*M* = 4.08, *SE* = 0.09); *t*(47) = 12.00, *p* < 0.001) and black hands (*M* = 3.81, *SE* = 0.11), *t*(47) = 7.13, *p* < 0.001). By contrast, in the asynchronous condition, scores did not differ from the midpoint for either the white hands (*M* = 3.16, *SE* = 0.21), *t*(46) = 78.00, *p* = 0.44), or black hands (*M* = 3.03, *SE* = 0.22), *t*(46) = 0.14, *p* = 0.89). The main effect for rubber hand color was also significant, *F*(1,91) = 7.65, *p* = 0.007, η2 = 0.08, indicating that the magnitude of the illusion was generally larger for the white RH as compared to the black one.

However, this analysis of self-reported RHI did not produce a significant Stimulation Condition x Rubber Hand Color interaction, *F*(1,91) = 0.89 *p* = 0.35, η2 = 0.01. The lack of an interaction might have been due to the slightly larger reported illusion for white than black hand illusion in the asynchronous condition, despite that neither hand in this condition is different significantly from the scale midpoint. Nevertheless, given our theoretical interests and predictions, and the patterns reported above, we tested the simple effects of race separately in the synchronous and asynchronous conditions. We found a significant effect between white and black RH in the synchronous condition (*t*47 = 2.61, *p* = 0.01), indicating stronger self-reported RHI for the white hand compared with the black hand. This difference was not significant in the asynchronous condition (*t*46 = 1.30, *p* = 0.20).

Finally, no significant effects were found for order, *F*(1,91) = 0.21, *p = *0.65, η2 = 0.002, the interaction of order and stimulation condition, *F*(1,91) = 0.72, *p* = 0.40, η2 = 0.008, the interaction of order and rubber hand color, *F*(1,91) = 1.56, *p* = 0.22, η2 = 0.02, the interaction of stimulation condition and rubber hand color, *F*(1,91) = 0.89, *p = *0.35, η*2* = 0.01, or the three-way interaction, *F*(1,91) = 1.50, *p* = 0.22, η*2* = 0.02.

#### Correlations

Based on our predictions, Pearson correlations were performed between participants’ characteristics (as measured by IAT and IRI) and RHI measures (onset time of the RHI, proprioceptive drift and RHI questionnaire) in the synchronous condition. Given the absence of RHI in the asynchronous stimulation, as theoretically expected, we did not pursue further correlational and meditation analyses for this condition.

Consistent with our hypothesis, onset time of the RHI for the black RH was significantly correlated with IAT score (r = 0.31, *p* = 0.03), such that participants with higher implicit anti-Black bias required more time to perceive the illusion with the black RH (see Table 2). IAT scores were not directly correlated with proprioceptive drift or self-report indices of the RHI for the black RH, nor any assessments of the RHI for the white RH. Finally, no significant correlation was found between IRI and RHI scores.

**Table 2:** Correlation analyses between IAT, IRI and RHI measures.

	White Rubber Hand			Black Rubber Hand		
	Time	Drift	Quest.	Time	Drift	Quest.
IAT	0.02	−0.26	0.03	0.31*	−0.06	−0.09
Perspective-Taking	−0.21	−0.11	−0.04	−0.0003	−0.14	0.02
Empathic concern	0.06	−0.23	−0.02	−0.11	0.13	0.11
Fantasy	−0.05	−0.15	−0.11	0.02	−0.09	−0.09
Personal distress	0.26	−0.11	−0.09	0.14	−0.04	−0.01

*p < 0.05.

Additionally, correlation analyses were also performed between the three RHI measures. As presented in Table 3, we found significant correlations between time to illusion, proprioceptive drift, and magnitude of illusion (as measured by the questionnaire) for the black RH but not for the white RH.

**Table 3:** Correlation analyses between RHI measures.

Correlations	White rh	Black rh
TIME, DRIFT	−0.02	−0.41**
TIME, QUEST	−0.07	−0.56**
DRIFT, QUEST	0.31*	0.40**

### Mediation Analysis

In order to explore the possibility that implicit prejudice effects on the rubber hand illusion occur, at least in part, through differences in time to illusion, a moderated mediation analysis (Model 59) was conducted using the PROCESS macro^28^ in SPSS 20.0. IAT score was entered in our model as the independent variable and time to illusion as the mediator. We tested this model with two different outcomes: proprioceptive drift and illusion magnitude. Because our question regarding prejudice effects pertained only to effects on black RHI and not the white RHI, we included the RH color as a moderator in our model. Bootstraping analysis with bias correction was employed to address for conditional indirect effects and an Index of Moderator Mediation was derived to address the equality of these effects. This particular model was tested because RH color was presented during the illusion induction and therefore could have had moderating effects on time to onset, proprioceptive drift, and questionnaires.

**Figure 6:**
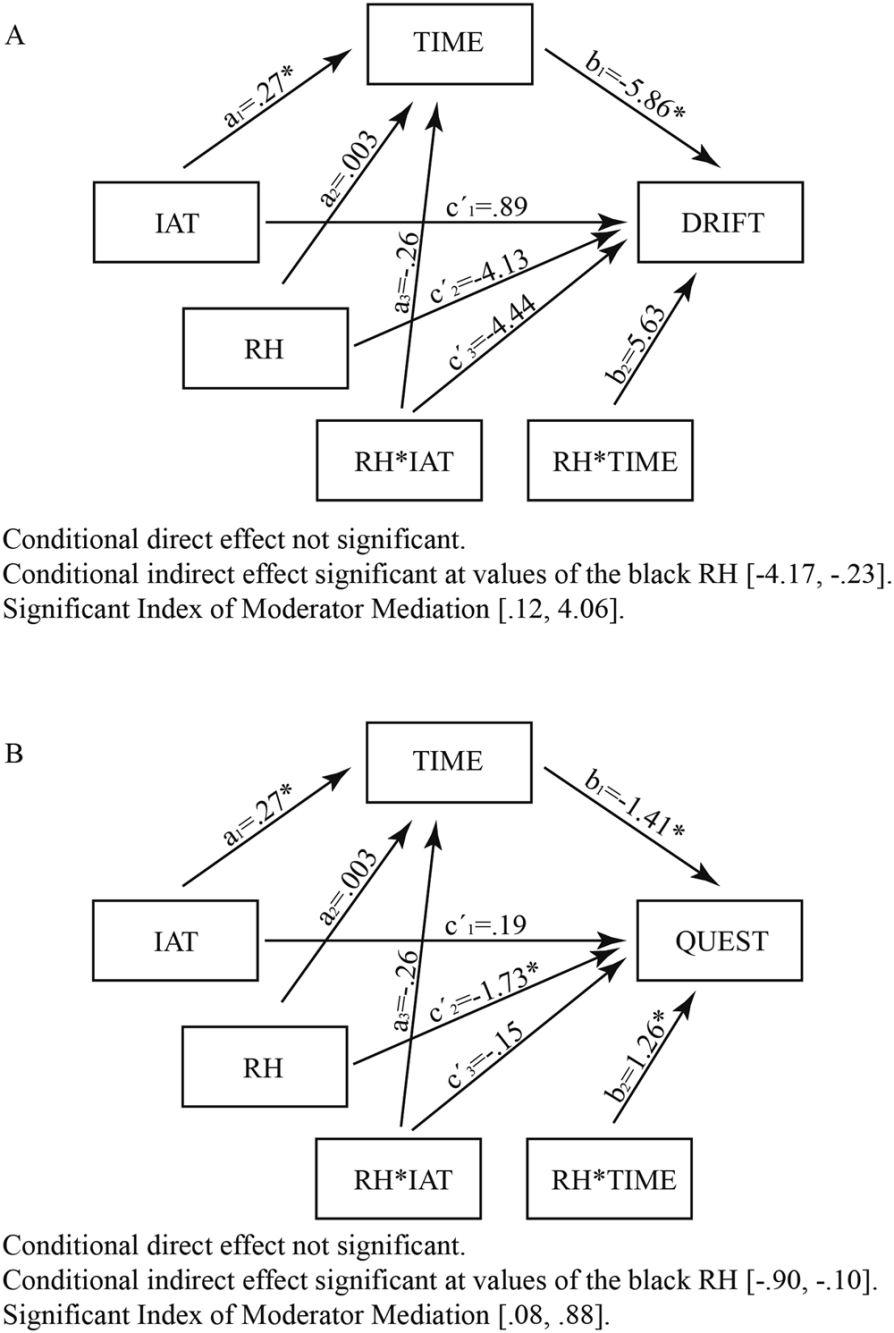
Moderated Mediation analysis with proprioceptive drift (**A**) and Questionnaire (**B**) as the outputs.

We first tested this model with proprioceptive drift as the outcome. Figure 6A presents the statistical diagram with the coefficients of all paths regarding the effects of IAT score on proprioceptive drift, via time to illusion, as moderated by RH color. A test of this model indicated that IAT score predicted time to illusion, B = 0.27, S.E. = 0.12, *t*(92) = 2.24, *p* = 0.03, and time to illusion predicted proprioceptive drift, B = −5.86, S.E. = 2.17, *t*(90) = −2.70, *p* = 0.008. The direct effect of IAT score on proprioceptive drift was not significant at values of the moderator (Black: B = 0.89, S.E. = 1.90, *t*(90) = 0.47, *p = *0.64; White RH: B = −3.55, S.E. = 1.81, *t*(90) = −1.97, *p* = 0.05). Importantly, however, a significant conditional indirect effect emerged for the black RH (CI: [–4.08, −0.21]) but not for the white RH (CI: [−0.65, 0.49]). Finally, we found a significant index of moderated mediation (Index = 1.59, S.E. = 1.00, CI: [0.10, 4.06]). Thus, these results show that IAT score predicted proprioceptive drift through the time to onset, in the black RH condition only.

Next, we tested this model with scores from the magnitude of illusion questionnaire as the outcome. Figure 6B presents the statistical diagram with the coefficients of all paths regarding the effects of IAT score on magnitude of illusion (Questionnaire) through time to illusion and moderated by the RH color. IAT score predicted time to illusion, B = 0.27, S.E. = 0.12, *t*(92) = 2.24, *p* = 0.03, and time to illusion predicted proprioceptive drift, B = −1.41, S.E. = 0.30, *t*(90) = −4.67, *p* < 0.0001. The direct effect of IAT score on magnitude of illusion was not significant at values of the moderator (Black: B = 0.19, S.E. = 0.26, *t*(90) = 0.72, *p* = 0.47; White RH: B = 0.44, S.E. = 0.25, *t*(90) = 0.17, *p* = 0.86). Importantly, again, a significant conditional indirect effect emerged for the black RH (CI: [−0.90, −0.10]), but not for the white RH (CI: [−0.10, 0.06]). Additionally, as it can be observed in Fig. 6B, Finally, we found a significant index of moderated mediation (Index = 0.38, S.E. = 0.19, CI: [0.07, 0.84]). Thus, these results show that IAT score predicted the magnitude of the illusion through the time to onset moderated by the black RH.

## Discussion

The aim of this research was to determine whether outgroup racial cues interfere with the time to perceptually incorporate the hand of a different-race person, and whether this interference relates to implicit prejudice. We tested whether the onset of the rubber hand illusion is moderated by the skin color of the rubber hand (i.e., white vs. black prototypic hands), and whether the effect was associated with the participants’ implicit racial prejudice. We hypothesized that the onset time would be greater for black RH as compared to the white RH and would function as a mediator between IAT score and the quality of the illusion.

As hypothesized, participants took more time to experience the black RH as their own compared with the white RH. Moreover, the black RH produced lower intensity values of embodiment, as well as smaller body proprioceptive drift, compared with the white RH. We also observed a significant correlation between the onset time of the illusion induced by the black RH and IAT score (i.e., the higher the implicit racial bias, the longer the time to perceive the illusion). To further comprehend the links between these variables, mediation analysis was conducted. We found significant indirect effects of IAT score on both proprioceptive drift and magnitude of illusion through onset time of illusion only for the black RHI. Therefore, our findings extend previous literature by showing that another measure – onset time of the illusion – elucidates the link between baseline implicit prejudice and the quality of the RHI. The knowledge of the temporal dynamics in processing and creation of percepts (such as the RHI) is crucial to a better description of the effect and development of dynamic process models.

### Self-reported measures

Consistent with previous studies, we observed a general effect on self-reported measures of the RHI with synchronous stimulation regardless of skin color. As shown previously, the magnitude of the illusion is reduced when the object differs in terms of shape from one’s own body-part^29–32^, but not when it differs in terms of color^20^. Indeed, with visuo-tactile multisensory integration, body ownership can be induced even when the skin color is from another ethnic group^12,17^. However, the effect on self-reported measures of the illusion was stronger for the white RH as compared to the black RH.

Our results showed that the illusion magnitude was higher when the RHI was induced by the white RH. Similar results were reported by Farmer *et al*.^12^, showing that white participants reported stronger RHI as measured by the questionnaire for the white RH when compared to the black RH. Skin color is indeed a key element in the psychological representation of different ethnic groups, both on the categorization and the automatic activation of implicit associations attributed to each group^33^, supporting the interpretation that skin color effects represented social group membership.

### Proprioceptive drift

Besides the effect on self-reported measures, we also found significant differences between white and black RH conditions on proprioceptive drift measures. This is in contrast with previous literature as Farmer and colleagues^12^ did not find significant effects on behavioral measures such as proprioceptive drift. This is an important difference between both studies, as proprioceptive drift might be seen as a more objective illusion index. When comparing the proprioceptive drift between the two studies, we can see that Farmer *et al*.^12^ found a drift of approximately 1.4 cm for the white RH, whereas we found a drift of 4.3 cm. With regard to the black RH, Farmer *et al*.^12^ found a drift of 2.2 cm, which was closer to the drift we observed for the same condition (2.3 cm). Looking at the literature on the RHI, we see that drifts tend to increase gradually over time of stimulation with values reaching about 4 cm by the end of the first minute^34^. Therefore, compared with our study as well as previous studies, Farmer *et al*.^12^ observed small proprioceptive drifts for both the white and the black RH. Thus, their lack of difference between white and black RH might be due to this general small effect on drift. Several methodological differences may help to elucidate the different findings. In our study, we used an electronic device perfectly controlling for angle, speed and distance. Also, highly realistic black and white hands were achieved with a specialized prosthetic in different versions considering skin color and gender. Thus, our procedures induced higher proprioceptive drift that in turn allowed the observation of difference between white and black RH. In Farmer *et al*.^12^, this difference between RH color on drift was prevented by low proprioceptive drift observed in both white and black RH.

Finally, to explain our findings on drift, it is important to understand what is behind the typical proprioceptive drift. The typical drift observed in RH studies is due to the integration of the visual-tactile information and its mental representation. The drift relies upon an attempt to coherently integrate the RH with the body-schema in a way that an action could be correctly implemented. In our study, the displacement induced by the black RH was 44% smaller than the displacement induced by the white RH. In this scenario, the reduced proprioceptive drift observed for the black RH may be revealing of the difficulty of fitting this hand into the participant’s white-represented body schema.

### Onset time of illusion

An important contribution of the present research is the measurement of onset time to illusion—an assessment of the RHI that has not been used in previous studies. We found that the incorporation of the black RH took significantly more time when compared with the incorporation of the white RH. These data provide new information on the processing speed of visual-tactile integration when there is incongruence between the visual information and the body model, providing further insight into the mechanisms involved in the process of intergroup body perception.

The inclusion of the onset time in theoretical models of body ownership enables the comprehension of this phenomenon in terms of its hierarchical processing of construction. In the neurocognitive model of the RHI proposed by Tsakiris *et al*.^7^, the initial stage of the illusion involves a comparison between the object’s visual input and one’s own body model. In our experiment, the visual input was found to delay the embodiment of the black RH. It is possible that this delay is due to a more demanding task of fitting a distinctly different-colored hand into one’s body scheme. These findings suggest that the illusion is much more than a bottom-up process of integrating visual and tactile information. Rather, RHI seems to also involve a top-down process associated with internal models of body and self, consistent with other recent work showing that race may have top-down effects on relatively low-level perceptual processes^2,35,36^.

### Racial bias effects on RHI

The mediation analysis revealed interesting associations between racial bias and race embodiment. For the white RH, we found neither direct nor indirect (through the onset time of illusion) effects of IAT scores on the magnitude of the illusion or proprioceptive drift. This lack of effects suggests that the multisensory integration of the white RH is more automatic and less vulnerable to other variables than the integration of the black RH. However, it is interesting to note that for the black RH, we found a significant indirect effect of IAT score on the magnitude of the illusion and the proprioceptive drift, as mediated by onset time of illusion. No direct effects of IAT score on the magnitude of the illusion or the proprioceptive drift were found without the inclusion of the mediator. This model shows how implicit prejudiced associations influenced multisensory processing and, therefore, the quality of the illusion. Thus, the multisensory integration of the black RH is influenced by implicit prejudice as indexed by the onset time of the illusion, which acts as a key element linking implicit bias with the magnitude of the illusion and the proprioceptive drift. The observed indirect effect suggests that an implicit association may influence the time to integrate different modal inputs (in this case, visual and tactile) in unique percepts.

Therefore, we found that greater prejudice was associated with a shorter experience of the illusion, which in turn resulted in less proprioceptive drift and a lower reported experience of the magnitude of illusion. Further experiments should test whether longer synchronous stimulation would reduce the difference between the black and the white RHI.

More broadly, our findings add to the growing body of research on intergroup embodiment, which suggests that prejudices operate not only in the mind, but also in the way we perceive and relate to other people physically. Although the notion of intergroup multisensory integration is still very new, it offers a novel perspective on prejudice and intergroup relations that is likely to generate new advances. For example, the present finding that implicit prejudice was associated with the RHI for the black hand suggests the possibility that intergroup effects on multisensory integration may represent a previously-unexamined component of implicit attitudes.

### Empathy

Despite some previous evidence for a link between empathy and the RHI^37,38^, we did not find a significant effect of empathy on the RHI. For example, a recent study by Seiryte and Rusconi^39^ demonstrated that empathy predicts the strength of the RHI. However, in this study the authors selected volunteers from the two extremes of empathy – top and bottom quintiles on the Empathy Quotient – allowing for direct comparisons between two groups. By contrast, in our study, the IRI values were normally distributed and in the normal range. This suggests that individual differences in empathy may relate to RHI at the extremes, but perhaps not across the typical range.

### Limitations

The present results should be understood in light of some possible limitations. One possible limitation may be associated with the use of an IAT version containing only male pictures, while our participants were both male and female. As in much prior research using the IAT, male faces are often used because Black men are more strongly associated with prejudices and stereotypes associated with Black people compared with Black women. For this reason, the use of Black male faces in the IAT may provide a stronger assessment of anti-Black implicit attitudes. Future experiments should test the effects of IAT with pictures from both genders. To partially control this limitation, we performed independent t-tests considering the gender of the participants. No significant differences between male and female volunteers in terms of IAT performance were found for both stimulation conditions: synchronous (t(46) = 0.72, *p* = 0.48) and asynchronous (t(28) = −0.51, *p* = 0.61). Additionally, the use of a stopwatch to register the onset time of illusion could also have interfered with accurate measurements (i.e., demand effects). The use of audio recording could provide an alternative and more precise measure.

We did not include a third RH color and, therefore, a possible interpretation of the current results is that the effects were due to the mere discrepancy between the skin colors of the RH and participants’ hand, independently of any ethnic/racial associations. However, we believe this is unlikely; the significant indirect effect found in the mediation analysis suggests that racial bias is largely driving the delay in multisensory integration for the black RH. Also, our hypothesis of an effect of racial bias in multisensory integration finds some support in other research paradigms. For example, Avenanti, Sirigu and Aglioti^13^ investigated the effect on participants’ sensorimotor resonance while observing videos with painful content (a needle being applied to a hand). The sensorimotor resonance was indexed by measures of motor evoked potential (MEP) amplitude as assessed by the use of transcranial magnetic stimulation. The authors found that white participants presented a reduction of MEP amplitude (i.e., sensorimotor resonance response) when observing the induction of pain in white and purple colored hands when compared with the black hand. Thus, the effect appears to be specific for skin colours with specific semantic associations and attributable to existing racial/ethnic outgroups. Even so, future studies with another RH colors (in addition to white and black) may help to further clarify this interpretation.

## Conclusion

In summary, our findings help to reveal how the temporal dynamics of self-other bodily overlap are affected by implicit prejudice and how this interference affects the illusion perception. The study of the onset time of illusion as well as its temporal dynamics is crucial, as delays in multisensory integration might impact our affective evaluation and agency attribution to others as well as how we are socially connected to them.
